# A Review of Selected Factors of Salivary Gland Tumour Formation and Malignant Transformation

**DOI:** 10.1155/2018/2897827

**Published:** 2018-08-01

**Authors:** Pawel Sowa, Karolina Goroszkiewicz, Joanna Szydelko, Joanna Chechlinska, Katarzyna Pluta, Wojciech Domka, Maciej Misiolek, Wojciech Scierski

**Affiliations:** ^1^Department of Otorhinolaryngology and Oncological Laryngology in Zabrze, Medical University of Silesia, Katowice, Poland; ^2^Department of Otorhinolaryngology, Faculty of Medicine, University of Rzeszow, Poland

## Abstract

Salivary gland tumours represent about 6% of head and neck neoplasms and about 0.5% of all malignancies in humans. Tumour growth and malignant transformation are complex processes involving various actions of molecules. Furthermore, some malignant salivary gland tumours are deemed to be caused by dedifferentiation or malignant transformation of benign tumours. The mechanisms of this transformation depend on a variety of different elements, such as cell cycle regulators, oncogenes, proteins, angiogenesis factors, and adipocytokines. The authors used PubMed, Medline, and Google websites to find and review the most significant papers related to malignant transformation in benign salivary gland tumours.

## 1. Introduction

According to the World Health Organization (WHO), salivary gland tumours comprise about 6% of head and neck neoplasms and about 0.5% of all malignancies in humans. The global annual incidence is estimated between 0.4 and 13.5/100000. Mortality depends on the stage and type of the lesion; however, the 5-year survival rate is estimated at 72% [1]. The most common location of salivary gland tumours is the parotid gland (up to 80%), followed by the submandibular gland (10-15%) and the sublingual gland with minor salivary glands. Fortunately, benign tumours constitute about 80% of those pathologies.

Salivary gland tumours are a heterogenous group of pathologies. The WHO classification system (2005; modified in 2017) recognizes benign tumours and primary and secondary malignant tumours, depending on the origin (see Table 1) [2, 3].

The increased incidence of salivary gland tumours has recently been observed in our department and other friendly clinics around Europe. With the increase in the total number of salivary gland tumours, the number of malignant pathologies also increases.

Some salivary gland carcinomas originate from dedifferentiation or malignant transformation of benign tumours. The mechanism of malignant transformation is a complex process, depending on many different factors. Moreover, although malignant transformation of benign salivary gland tumours is a known phenomenon, the mechanisms of this process are not well understood.

In this paper, we present five groups of parameters that may play a crucial role in formation of salivary gland tumours and their malignant transformation:

- cell cycle regulators, including cyclines, tumour suppressors, and transcription factors,
- oncogenes—we present a few that can be characteristic of a specific tumour type,
- proteins, including *β*-catenin, defensins, tenascin, and mucins,
- parameters of angiogenesis where the vascular endothelial growth factor (VEGF) and CD105 are discussed,
- adipocytokines—leptin, ghrelin, and adiponectin.

We believe that the present paper may bring an interesting view and result in further research related to salivary gland pathologies and oncology.

## 2. Materials and Methods

The PubMed, Medline, and Google websites were used to search and review the most significant papers on the topic of the mechanism of malignant transformation in benign salivary gland tumours. No particular exclusion or inclusion criteria were used for the selection of the articles for this paper. This review highlights five important groups of factors associated with malignancy.

The key words used during the review of the literature were as follows: “salivary gland tumour marker”, “salivary gland cell cycle”, “salivary gland oncogenes”, “salivary gland proteins”, “salivary gland angiogenesis”, and “salivary gland adipocytokines”. In total, 321 articles were reviewed. The articles published in journals in Journal Citation Reports were most desirable.

## 3. Results and Analysis

### 3.1. Cell Cycle Regulators

The cell cycle regulation includes different processes which are necessary for cell survival, such as the prevention of uncontrolled cell proliferation. A few classes of molecules are involved in this action. Cyclins and cyclin-dependent kinases (CDKs) lead to the progression of the cell cycle, while CDK interacting protein/kinase inhibitory protein (Cip/Kip) and inhibitor of kinase 4/alternative reading frame (INK4a/ARF) cause the inhibition of cell cycle progression (“tumour suppressors”). Furthermore, transcription factors are also involved in the cell cycle regulation.

Expression of G1-phase cell cycle regulators is commonly dysregulated in human malignant tumours. Etges et al. [4] investigated the expression of different cell cycle regulators in normal salivary glands and salivary gland tumours, i.e., pleomorphic adenoma (PA), adenoid cystic carcinoma (ACC), mucoepidermoid carcinoma (MEC), epithelial-myoepithelial carcinoma (EMC), carcinoma ex pleomorphic adenoma (CXPA), malignant myoepithelioma (MEM), and polymorphous low-grade adenocarcinoma (PLGA). The majority of tumour samples demonstrated overexpression of CDK-4, P16 and E2F-1. However, the expression of retinoblastoma protein (pRb) was decreased. A similar study was conducted by Patel et al. [5] and revealed that cyclin D1 and p16 were more likely to be expressed in neoplastic tissues than in normal epithelial and stromal components of PA and CXPA. However, other studies demonstrated that the overexpression of cyclin D1 probably does not play a significant role in the pathology of benign and malignant salivary gland tumours [6, 7].

Affolter et al. [8] compared the expression of p21, p27, and p53 (which belong to a tumour suppressor family) in benign and malignant tumours of salivary glands. Their study results revealed that 78% of all ACCs had no sign of the expression of p27; however, in 60% of the adenocarcinomas the expression level was increased. The expression of p21 was found in all malignant tumours.

As previously mentioned, the p53 contributes to malignant transformation and is used as a diagnostic marker for malignancy [9]. However, DeRoche et al. showed its expression in benign PA. Those authors used immunohistochemical stains for p53, AR, and HER-2/neu in 41 histologically and clinically benign PAs. Therefore, these markers cannot be used in predicting early malignant transformation [10].

According to Maruya et al., p63, particularly DeltaNp63L isoform, seems to be involved in malignant transformation of salivary glands. Staining with DeltaNp63 was negative or slightly positive in most benign neoplasms, but highly expressed in ACC, MEC, and myoepithelial carcinomas. The second investigated isoform, i.e., TAp63, was highly expressed in benign tumours and negative in most carcinomas [11].

Bub1 is a cell cycle regulator important in the establishment of the mitotic spindle checkpoint. Disturbed mitotic checkpoints were reported in different neoplasms. Mutations or altered gene expression of Bub1 has been investigated in various pathologies such as colon, esophageal, lung, gastric, and breast tumours or melanoma [12–17]. Shigeishi et al. [18] examined the expression of Bub1 in the normal salivary gland and in benign and malignant tumours. Their study revealed an increased level of Bub1 expression in malignant tumours.

Furthermore, the signal transducer and activator of transcription 3 (STAT3) is constitutively active in different types of malignant tumours. Araujo et al. [19] compared its activity in the normal salivary gland tissue, pleomorphic adenoma, and malignant tumours. The expression of STAT3 and STAT3P (Phospho-STAT3) in PA was similar to the expression in the normal tissue. In malignant tumour cells, unlike in PA and the normal tissue, STAT3 was present in the nuclei, which was significant and most evident in ACC. The presence of STAT3 in the nuclei may play an important role in oncogenesis, probably by stimulating tumour cell proliferation and blocking apoptosis.

### 3.2. Oncogenes

Oncogenes arise from proto-oncogenes, normally involved in cell division. The mutation that occurs in a proto-oncogene transforms it into an oncogene. The oncogene product remains constantly in the active form leading to the uncontrolled proliferation. In tumour cells, these genes are highly expressed or mutated. Thus, targeted therapies (anti-HER2, anti-EGFR, etc.) may become useful tools in the treatment process in particular patients.

Cros et al. assessed C-KIT, EGFR, HER2, MUC1, phospho-mTOR, androgen/estrogen/progesterone receptors, and Ki67 expression in different types of salivary gland malignancies. They reported high levels of HER2 and androgen receptors in salivary duct carcinomas, C-KIT in myoepithelial carcinomas, and EGFR in mucoepidermoid carcinomas [20].

A few highly specific and pathognomonic proto-oncogenes have recently been found among salivary gland tumours, i.e., mucoepidermoid carcinoma translocated-1 (MECT1) for mucoepidermoid carcinoma [21, 22], MYB for adenoid cystic carcinoma [23, 24], EWSR for hyalinizing clear cell carcinoma [25], or ETV6 for mammary analogue secretory carcinoma [26]. Chromosomal translocations result in creating fusion oncogenes from the above-mentioned proto-oncogenes. These fusion oncogenes may be often derived from, and encoded for, transcription factors, transcription regulators, and receptor tyrosine kinases that are frequently involved in oncogenesis [27].

### 3.3. Proteins

#### 3.3.1. *β*-Catenin

*β*-catenin acts as a structural protein at cell-cell adherens junctions and also as a transcriptional activator mediating Wnt (Wingless-type) signal transduction [28]. The Wnt/*β*-catenin signaling pathway regulates key processes in cell proliferation, differentiation, and also oncogenesis. Alterations and hyperactivation of this pathway promote tumour development in numerous tissues, including salivary glands [29–32].

Prado et al. [33] revealed that *β*-catenin accumulation in the cytoplasm may lead to malignant transformation of PA into CXPA.

*β*-catenin, *α*-catenin, and E-cadherin form complexes to establish a link with the actin cytoskeleton. Shibuya et al. revealed that abnormal cadherin-catenin complexes in ACC may not act properly, which leads to the dissociation of cells in salivary gland tumours and subsequently results in local invasion and metastasis [34].

In the study by Chandrashekar et al. [35], the presence of *β*-catenin was detected in 92.3% of benign tumours and in 62.5 % of malignant tumours. Those authors proposed that decreased cell membrane expression of *β*-catenin and its predominant cytoplasmic accumulation could be responsible for malignant transformation.

Genelhu et al. [36] assessed the location of *β*-catenin in the tissues of PA and CXPA. The study revealed membranous *β*-catenin staining in the normal gland parenchyma. In PA tissues, the distribution of *β*-catenin was irregular, occasionally with membrane/cytoplasmic localization. Unexpectedly, the nuclear expression of *β*-catenin was present in an area of solid cell proliferation of PA in 1 case, which might suggest a possible association of this molecule with early molecular events. Membrane/cytoplasmic localization was observed in well-differentiated CXPA. The cytoplasmic/nuclear expression was present in poorly differentiated tumours. Genelhu's data suggested that *β*-catenin may play an important role in differentiation and transition to the malignant phenotype of CXPA.

#### 3.3.2. Defensins

Human beta defensins (hBD) 1, 2, and 3 are peptides with antimicrobial potency due to their ability to disintegrate membranes. They are involved in the formation and progression of PA and malignant salivary gland tumours [37]. It was shown that, in these tumours, hBD-1 might act as a tumour suppressor, whereas hBD-3 might be a proto-oncogene [38].

Wenghoefer et al. [37] conducted a study which involved immunohistochemical examination of the location of hBD-1 in salivary gland cells. HBD-1 was located in the cytoplasm in benign salivary gland tumours and in the healthy tissue. It was shifted from the cytoplasm to the nucleus in malignant tumours. This study confirmed that the hBD-1 gene may play a role as a suppressor gene and also revealed that nuclear accumulation of this protein could implicate the malignant progression of salivary gland tumours.

Winter et al. [39] compared the expression of human *α*-defensins (DEFA) in different benign and malignant histological types of salivary gland tumours with the healthy tissue. In comparison with the healthy tissue, the gene expression of DEFA 1/3 and 4 was significantly increased in all tumours except for PA in which decrease of DEFA 4 was evident. Another characteristic feature of PA was only a slight increase of DEFA 1/3 expression, compared with a prominent increase in other types of the tumour tissue. A decreased gene expression of DEFA 1/3 and 4 might protect PA from malignant transformation.

Another study by Meng et al. focused on hBD-2 whose expression was significantly increased in benign salivary gland tumour tissues and also in inflamed salivary glands [40]. Chronic inflammation is known to be a trigger of tumour development or malignant transformation. Leukocytes, whose elevated level is associated with chronic inflammation, release inflammatory mediators such as arachidonic acid, chemokines, and cytokines. Permanent exposure to these factors results in cell proliferation, angiogenesis, or oncogene activation. All these processes are strongly associated with salivary gland tumour formation and malignant transformation as described in this paper [41].

#### 3.3.3. Tenascin

Tenascin is an extracellular glycoprotein and its deposition in the tumour stroma was found in various pathologies, e.g., breast, prostate, colon, stomach, lung, skin, laryngeal, ovarian, and uterine cancers. Its expression was correlated with tumour morphogenesis as well as with local invasiveness and tumour metastatic behaviour [42, 43].

Tenascin deposition was reported to be involved in the mechanisms of malignant transformation of PAs into carcinomas [44, 45]. Felix et al. [44] demonstrated that the content of tenascin was higher in malignant and benign stromal areas of CXPAs as opposed to similar areas of PAs.

#### 3.3.4. Mucins

Mucins are a family of glycoproteins which in spite of being a constituent of mucus are involved in signaling pathways responsible for coordinating cell proliferation, differentiation, and apoptosis [46].

Alterations in the distribution and expression level of mucin proteins were reported in salivary gland neoplasms. The expression patterns of particular types of mucins may correlate with the histopathological type of the tumour. Increased expression of mucin 1 (MUC1) may also result in malignant transformation of PA into CXPA [46–48].

### 3.4. Angiogenesis

Tumour angiogenesis is a complex process of blood vessel growth, induced by tumour itself and by the secretion of multiple growth factors, e.g., VEGF. These factors induce capillary growth into the tumour mass, thereby allowing for its nutrition, progression, invasiveness, and metastasis. Targeting angiogenic factors has become an effective way of the inhibition of tumour growth in different types of cancers [49].

The increased expression of VEGF in salivary gland neoplasms was reported in a few studies. This observation may account for tumour progression, lymph node metastases, and worse survival rates [50–52].

Furthermore, CD105 (endoglin) is a marker for neoangiogenesis [53]. It is a receptor for TGF*β* signaling and plays a pivotal role in angiogenesis. It is important for endothelial cell proliferation, thereby promoting the activation phase of angiogenesis. Moreover, increased expression of CD105 is a feature of newly formed tumour blood vessels and positively correlates with the risk of metastasis [54–56].

Soares et al. [57] assessed tumour vascularization in histological samples of early CXPA, advanced CXPA, and PA by measuring the total microvascular area and microvessel density (MVD) using CD34 and CD105 antibodies. During the adenoma-carcinoma sequence (PA without malignant transformation, early CXPA, widely invasive CXPA), the MVD for CD105 gradually increased. The strong correlation between MVD and tumour progression suggests that the angiogenic switch is required during the malignant progression of PA into CXPA. Moreover, in minimally invasive carcinoma, CD105-positive vessels were more numerous compared to PA without malignant transformation, suggesting that in these early malignant lesions angiogenesis had already been activated. This finding is in agreement with a model in which angiogenesis was found to be activated early during the multistage development of invasive cancer, as was revealed in histological analyses of premalignant, noninvasive lesions arising in a variety of organs [58].

In addition, another factor, i.e., Epidermal Growth Factor Receptor (EGFR), is considered crucial in tumour angiogenesis, especially in ACC [59, 60]. In the study of Wang et al. [61], the expression of EGFR, CD31 (a marker for the presence of blood vessels), CD146 (molecule highly expressed in pathological vessels), and the hypoxia-inducible factor-1*α* (HIF-1*α*; main transcription factor involved in angiogenesis) was evaluated in the normal salivary gland tissue, PA, and ACC. A higher EGFR expression was observed in ACC and it positively correlated with the expression of HIF-1*α*, CD146, and CD31. These results suggest that EGFR may be related to angiogenesis by affecting the expression of HIF-1*α* and CD146.

Furthermore, promising research on Mena (mammalian Ena), belonging to a protein family regulating the actin cytoskeletal network, was presented a few years ago by Gurzu et al. Altered expression of this protein can be associated with metastasis. The expression of Mena was analysed in normal salivary glands and in benign and malignant tumours and correlated positively with tumour grade. Therefore it can be considered a tumour marker. Additionally, the expression of Mena was demonstrated in some stromal endothelial cells, thereby being important also in tumour angiogenesis [62].

### 3.5. Adipocytokines

#### 3.5.1. Leptin

Leptin is a cytokine, which was originally reported to play a key role in the regulation of food intake and energy expenditure. However, a few studies revealed that it can also be found in human salivary glands. Autonomous expression of leptin and its receptors by salivary gland cells independently of adipocytes was demonstrated [63–65]. Recent studies revealed that leptin is involved in the processes of tumourigenesis [66].

Schapher et al. in their study showed that, in all PAs, lymphadenomas and carcinomas leptin was expressed in much higher amounts than in healthy salivary gland tissues. Their PCR studies revealed that leptin was not carried by the bloodstream but was produced by tumour cells [67].

Moreover, the level of salivary leptin was significantly higher in women than in men, which implies the necessity to consider the gender of patients when leptin level is used as a potential tumour marker. However, serum leptin concentration may not discriminate between benign and malignant processes [68].

#### 3.5.2. Ghrelin

Ghrelin, a cytokine which is antagonistic to leptin in the control of satiety, was also found in saliva. It was shown that ghrelin was present in healthy salivary glands, but was absent in neoplastic tissues [69, 70]. Moreover, it was reported that ghrelin may affect tumour cells in a different manner, induce, or inhibit proliferation, depending on the tumour type, and its role still remains unclear [71]. Nevertheless, ghrelin is also known to be used for the treatment of chemotherapy-induced gastrointestinal side effects [72, 73].

#### 3.5.3. Adiponectin

Adiponectin, another adipocytokine, is a protein involved in numerous metabolic processes, mainly glucose level regulation and fatty acid oxidation.

In the study of Schapher et al., adiponectin ApM-1 was selected as a specific adipocyte marker. PCR analysis revealed that ApM-1 levels were significantly lower in tumours than in the normal tissue, which indicates that adipocytes were absent in salivary gland tumours [67].

Another study revealed that adiponectin serum levels were significantly higher in benign salivary gland tumours compared to malignant ones, which may suggest that this molecule may prevent malignant transformation [74].

Moreover, adipocytokines play an important role in the inflammatory process. As in the case of the previously mentioned defensins (hBD-2), some of the adipocytokines (e.g., leptin) act as proinflammatory factors inducing chronic inflammation, which may lead to carcinogenesis. Abnormal accumulation of adipose tissue in obese patients is considered to be an important tumour risk factor [75, 76].

## 4. Discussion

The processes of tumour formation and malignant transformation are complex and involve a variety of molecules and multidirectional actions.

We considered the cell cycle regulators. Molecules such as p16, CDK-4, E2F-1, p21, and p53 are reported to be overexpressed in salivary gland tumours: p27 is overexpressed in adenocarcinomas but not in ACC; p63 is expressed differently depending on the isoform; Bub1 and STAT3 are overexpressed in malignant tumours; the role of the cyclin D1 remains unclear. The second group of factors were oncogenes. According to the literature, different types of tumours have their own specific oncogene, which creates opportunities for targeted therapies. Subsequently, we focused on some proteins that were reported to play a role in oncogenesis. Overexpression and accumulation of *β*-catenin may lead to tumour formation and malignant transformation. Defensins may also participate in tumour pathology; however, there are differences among subtypes. Some may act as proto-oncogenes, while others may act as suppressors. Tenascin and mucins are shown to be the factors of malignant transformation from PA into CXPA.

Angiogenesis is a relatively well-known process associated with tumour growth. Most important angiogenic factors are VEGF, CD105, EGFR, HIF-1*α*, CD146, CD31, and Mena. All these factors correlate positively with angiogenesis progression.

The increased level of leptin is reported in salivary gland neoplastic tissues, while ghrelin is expressed in the healthy tissue and is absent in tumours. However, those adipocytokines may act antagonistically. The level of adiponectin is significantly higher in the healthy tissue.

## Figures and Tables

**Table 1:** Histopathological classification of salivary gland tumours, according to WHO.

Epithelial tumours	
Malignant	Benign

(i) Acinic cell carcinoma	(i) Pleomorphic adenoma (PA)
(ii) Mucoepidermoid carcinoma (MEC)	(a) Metastasizing pleomorphic adenoma
(iii) Adenoid cystic carcinoma (ACC)	(ii) Myoepithelioma
(iv) Polymorphous adenocarcinoma (PAC) (before 2017: Polymorphous low-grade adenocarcinoma – PLGA)	(iii) Basal cell adenoma
(v) Epithelial-myoepithelial carcinoma (EMC)	(iv) Warthin's tumour
(vi) Clear cell carcinoma	(v) Oncocytoma
(vii) Basal cell adenocarcinoma	(vi) Canalicular adenoma
(viii) Sebaceous carcinoma	(vii) Lymphadenoma
(ix) Intraductal carcinoma	(a) Sebaceous lymphadenoma
(a) Low-grade cribriform cystadenocarcinoma	(b) Nonsebaceous lymphadenoma
(b) Salivary duct carcinoma	(c) Sebaceous lymph adenocarcinoma
(c) Salivary duct carcinoma, not otherwise specified	(viii) Ductal papilloma
(x) Oncocytic carcinoma	(a) Inverted ductal papilloma
(xi) Adenocarcinoma, not otherwise specified (NOS)	(b) Intraductal papilloma
(a) Cystadenocarcinoma	(c) Sialadenoma papilliferum
(b) Mucinous adenocarcinoma	(ix) Cystadenoma
(xii) Myoepithelial carcinoma	
(xiii) Carcinoma ex pleomorphic adenoma (CXPA)	
(xiv) Secretory carcinoma	
(xv) Carcinosarcoma	
(xvi) Squamous cell carcinoma	
(xvii) Large cell carcinoma	
(xviii) Lymphoepithelial carcinoma	
(xix) Sialoblastoma	

Other epithelial lesions	

(i) Intercalated duct lesions	
(a) Intercalated duct adenoma	
(b) Intercalated duct hyperplasia (IDH)	
(ii) Nodular oncocytic hyperplasia	
(iii) Lymphoepithelial lesions	

Soft tissue tumours	

(i) Haemangioma	
(ii) Lipoma	
(iii) Nodular fasciitis	

Haematolymphoid tumours	

(i) Hodgkin lymphoma	
(ii) Diffuse large B-cell lymphoma	
(iii) Extranodal marginal zone B-cell lymphoma	

Secondary tumours	

(i) Tumours which have metastasized to the salivary gland from another location	

## Additional Points

*Summary.* This article reviews selected factors that influence the development of salivary gland tumours and their malignant transformation. We believe that our paper will be helpful in focusing on interesting directions of investigations in the studies on salivary gland tumours.
